# Removal of a Tumor from the Hyoid Bone

**Published:** 1856-06

**Authors:** A Dunlap, C. N. Woodward


### REMOVAL OF A TUMOR FROM THE HYOID BONE.

By Dr. A. DUNLAP. Reported by Dr. C. N. WOODWARD.

MR. EDITOR:

Thinking perhaps, it might not be uninteresting to the readers of the Register, I will give you a case, and operation performed by A. Dunlap, M. D., of Ripley, Ohio, assisted by B. M., M. D., May 2, 1856.

The subject, a man 28 years of age, sanguine billious temperment, with a tumor of twenty years standing, fatty fibrous, deep seated in the neck, directly under the chin, impeding articulation, and deglutition, I placed the patient under the influence of chloroform; respiration natural, pulse retaining its regularity, and falling but slightly, patient perfectly quiet.

Dr Dunlap made an incission from the symphasis of the inferior maxillary, extending down the median line about three inches dividing the ligaments and superficial facia, then by separating the muscles through the interstices the tumor was disloged from its bed, finding it supplied by viscous veins, he passed a ligature around the pedicle and detached it from the hyoid bone completing the operation, closed the wound with adhesive straps. Patient revived, quiet and doing well.

May 3d, patient doing well, 4th dressed wound found it healing by first intention. Patient sits up says he feels well; speach somewhat improved, inclines to partake of nourishment walks out of his room. 9th day, patient rapidly recovering says he feels perfectly well, articulation much improved, swallows with ease, and every symptom indicates a perfect cure. Patient returns home.

